# Factors associated with the double burden of malnutrition among adolescents, National Adolescent School-Based Health Survey (PENSE 2009 and 2015)

**DOI:** 10.1371/journal.pone.0218566

**Published:** 2019-06-14

**Authors:** Júlia Caffé Oliveira Uzêda, Rita de Cássia Ribeiro-Silva, Natanael de Jesus Silva, Rosemeire L. Fiaccone, Débora C. Malta, Naiá Ortelan, Maurício L. Barrato

**Affiliations:** 1 Departamento Ciência da Nutrição, Escola de Nutrição, Universidade Federal da Bahia, Salvador, Bahia, Brasil; 2 Instituto de Saúde Coletiva (ISC), Universidade Federal da Bahia, Salvador, Bahia, Brasil; 3 Instituto de Matemática, Universidade Federal da Bahia, Salvador, Bahia, Brasil; 4 Universidade Federal de Minas Gerais – Escola de Enfermagem, Belo Horizonte (MG), Brasil; 5 Centro de Integração de Dados e Conhecimentos para Saúde (CIDACS), Instituto Gonçalo Moniz. Fundação Oswaldo Cruz – FIOCRUZ, Salvador, Bahia, Brasil; University 9 of July, BRAZIL

## Abstract

**Objective:**

To assess the sociodemographic factors associated with the double burden of malnutrition (DBM) among Brazilian adolescents.

**Methods:**

This was a descriptive study based on data from 59,637 and 10,770 students who participated in the National Adolescent School-Based Health Survey (PeNSE), 2009 and 2015 editions, respectively. Weight and height measurements were obtained to evaluate nutritional status. DBM was classified as follows: adolescents with high BMI-for-age and low height-for-age (BMI/A: Z-score > +1 and H/A: Z-score < -2). Sociodemographic data on the participants were also collected. A multinomial logistic regression analysis was used to detect associations of interest.

**Results:**

The prevalence of DBM in the 2009 and 2015 editions of the PeNSE was 0.4% and 0.3%, respectively. In the 2009 edition, the chance of DBM was lower among boys (OR = 0.60; 95% CI = 0.45–0.81) and higher among those over 14 years old (OR = 2.40; 95% CI = 1.80–3.20), living in the country’s north and northeast regions (OR = 2.01; 95% CI = 1.49–2.84), and from families with a low maternal education level (OR = 1.48; 95% CI = 1.07–2.04). In the 2015 edition, no significant associations were found regarding the DBM outcome.

**Conclusion:**

The results indicate the presence of socioeconomic inequalities in the occurrence of DBM in the 2009 edition of the PeNSE. Simultaneous interventions in the area of equity are necessary to prevent the advancement of nutrition-related problems.

## Introduction

The epidemiological transition phenomenon, particularly in the context of low- and medium-income countries (LMICs), has been accompanied by another process called the nutritional transition [1]. This phenomenon is characterised by important changes in the patterns of food consumption [1], [2] combined with an increase in sedentarism in the population [3]. This scenario is reflected in the nutritional status, including changes in mean height and body composition [4], [5]. The nutritional transition in LMICs has been marked by an accelerated decrease in malnutrition among children and adults, but still includes unsettling incidence rates among the poorest. Simultaneously, an increase in overweight and obesity has been observed in all life stages [6], [1]. Hence, apparently antagonistic health-related problems have come to exist simultaneously in the population.

The coexistence of stunting and overweight in the same child or adolescent reflects the poor quality of diet and morbidity in the first two years of life, followed by excess energy consumption at a later life stage [7], [8], [3]. This condition is due to underlying determinants to which the adolescents are exposed, such as unfavourable environmental, social and economic factors [9], [10]. It is known that, among countries with similar economic levels, those with higher levels of social inequality have lower levels of health [11].

In Brazil, the improvement of health indicators has not been achieved homogeneously by all population groups, contributing to the maintenance of a persistent burden of morbidity, even to problems in which reductions have been observed. For example, undernutrition is still prevalent among the poorest, difficulting this burden to be reduced below certain levels. Recently, overweight, obesity and diet-related NCDs are rapidly increasing in the country (14). However, the way that socioeconomic disparities relate to DBM are not very well understood yet (15). The double burden phenomenon has been very well documented at household and community level in the various life stages [12] [3], [13]. However, very little is still known on the DBM at individual level, especially among adolescents [14]. Therefore, the aim of the present study was to measure and evaluate the sociodemographic factors associated with this phenotype in adolescents who participated in the 2009 and 2015 editions of the National Adolescent School-Based Health Survey (Pesquisa Nacional de Saúde do Escolar—PeNSE), in Brazil.

## Methods

### Study design, population and sampling method (PeNSE 2009–2015)

Data from the PeNSE 2009 and 2015 editions were used for the present study. These are national surveys conducted by the Brazilian Institute of Geography and Statistics (Instituto Nacional de Geografia e Estatística–IBGE) in collaboration with the Ministry of Health, which aimed to identify and measure the risk and protective factors of health among Brazilian adolescents. The PeNSE data are public and can be accessed freely at the IBGE website (http://www.ibge.gov.br/). The PeNSE is a school-based epidemiological survey, and the target population is students from public and private Brazilian schools [15]. Each edition has peculiarities regarding the sampling process. In the 2009 edition [16], the study was limited to 9^th^ grade students in public and private schools located in the 26 Brazilian state capitals and in the Federal District. The schools were grouped according to their public or private status. The primary sampling unit was the school, and the secondary sampling unit was the class. Each stratum was subjected to systematic sampling by lottery with a probability proportional to the number of schools in the respective stratum (n = 63,411 respondents; valid sample n = 63,411). The PeNSE 2015 [17] was composed of two independent probabilistic samples (1 and 2). In the present study, only sample 2 was included, which is composed of students in the 13- to 17-year-old age range (from 6^th^ grade to the last grade of high school) of public and private schools located in the 26 state capitals of the five Brazilian macroregions and in the capital of Brazil (n = 16,608 respondents; valid sample n = 10,926). Further methodological details are described in the respective reports of each survey [16], [17], [15].

In all editions of the PeNSE, the respective projects were submitted to and approved by the National Commission for Ethics in Research (Comissão Nacional de Ética em Pesquisa–CONEP) under opinion n. 11.537/2009, n. 16.805/2012 and n. 1.006.467/2015 and were in agreement with Resolution no. 196 of October 10, 1996 from the National Health Council (Centro Nacional de Saúde–CNS).

### Data collection

Student-related data were collected via a self-administered questionnaire with electronic devices, i.e., personal digital assistants (PDAs) in 2009 and smartphones in 2015. The IBGE surveyors distributed the devices to the students on the day of the interviews and acquainted them with how to use the devices. In addition to the questionnaire, anthropometric measurements were also collected to calculate body mass index (BMI). In 2009 and 2015, data on student weight and height were collected. In 2009, this encompassed the entire sample, whereas in 2015, these data were only collected in the sample used in the present study. Further information on the measurements are available in the reports on the 2009 and 2015 editions of the PeNSE [16], [17].

### Anthropometric indicators

Weight and height were used to assess the adolescents’ nutritional status by means of the height-for-age (H/A) and BMI-for-age (BMI/A) indices as Z-scores. BMI is defined as the ratio between weight (kg) and height in metres squared (m^2^). The values proposed by the World Health Organization (WHO) for children and adolescents between 5 and 19 years of age were used as references [18]. Data were calculated with the programmes WHO AnthroPlus, v.1.0.4. Outliers were excluded from the analysis [i.e., H/A (Z-score < -6 or > +6) and BMI/A (Z-score < -5 or > +5)] [19].

The outcome of interest was ranked into the following four categories:

- Category 0 (reference): adolescents with normal BMI and height-for-age (BMI: Z-score ≤ +1 and H/A: Z-score ≥ -2);
- category 1 (stunting): adolescents with normal BMI-for-age and stunting (BMI: Z-score ≤ +1 and H/A: Z-score < -2);
- category 2 (overweight): adolescents with high BMI-for-age and normal height-for-age (BMI: Z-score > +1 and H/A: Z-score ≥ -2);
- category 3 (defined as a double burden of malnutrition): adolescents with high BMI-for-age and stunting (BMI: Z-score > +1 and H/A: Z-score < -2).

### Sociodemographic variables

For the present study, variables with similar questions in both editions of the PeNSE (2009 and 2015) were harmonised. These sociodemographic variables were as follows: age (≤ 14 years/ >14 years); sex (male/female); race/ethnicity (white/non-white); maternal education level (non-educated or incomplete primary education/completed primary education or incomplete secondary education/completed secondary education or incomplete higher education/completed higher education/did not know); school type (public/private); region (south, southeast and centre-west/north and northeast). The household assets and services index was based on the possession of a landline telephone, computer (desktop, laptop, tablet, etc.), car and/or motorcycle, number of bathrooms with a shower, home internet access, whether the adolescent had her own cell phone and whether the household had a housekeeper (at least three days a week). The household assets and services index was constructed according to a previously described method [20].

### Statistical analysis

The sample was characterized using a descriptive analysis for complex sample. The association between sociodemographic variables and nutritional outcomes was verified by the Pearson’s χ2 test. In order to identify the sociodemographic variables that are associated with the nutritional outcomes, Odds Ratio (OR) and 95%CI were obtained by multinomial logistic regression analysis. Fitted models were adjusted for all covariates, including age. To account for the complex sampling design, weighting was applied to the estimates be representative of the intended population. The *svy* command in Stata software version 12.1 (StataCorp LP, College Station, USA) was used to consider this effect.

## Results

**Fig 1:**
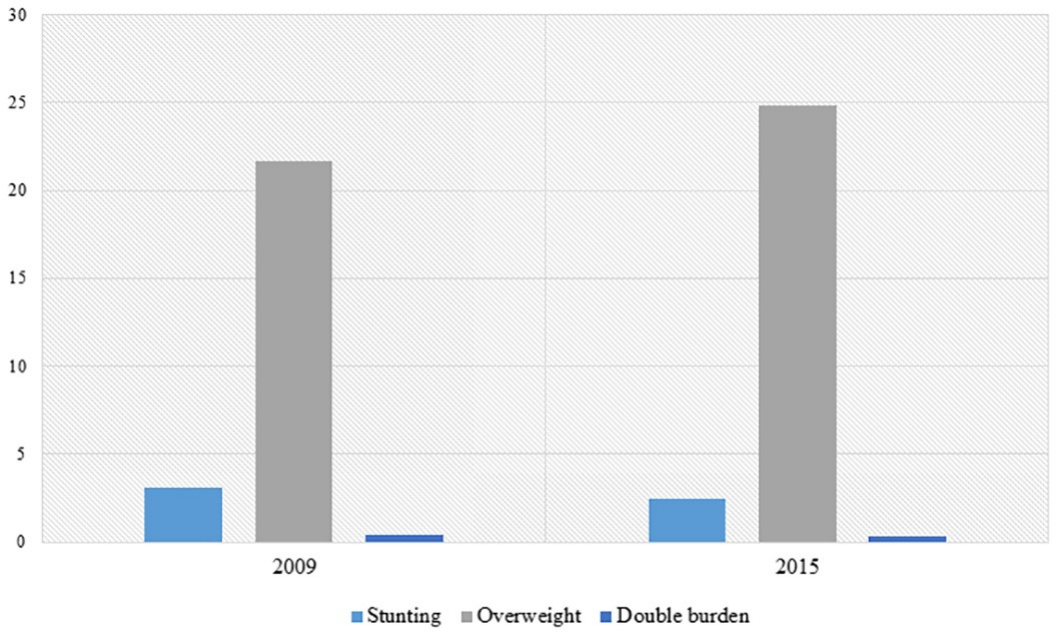
Prevalence of nutritional outcomes in adolescents. PeNSE (2009–2015). 2009 –Stunting (n = 1,582), overweight (n = 12,553) and double burden (n = 214). 2015 –Stunting (n = 2,631), overweight (n = 233) and double burden (n = 37).

The prevalence of overweight, stunting and DBM was 21.7% (n = 12,787; 95% CI 21.3–22.0), 3.1% (n = 1,819; 95% CI 2.9–3.2) and 0.4% (n = 214; 95% CI 0.32–0.41), respectively, in the PeNSE 2009. In the 2015 edition, the prevalence of the above outcomes was 24.8% (n = 2,668; 95% CI 24.0–25.6) for overweight, 2.5% (n = 270; 95% CI 2.2–2.8) for stunting and 0.3% (n = 37; 95% CI 0.23–0.45) for DBM (Fig 1).

The data from the PeNSE 2009 edition show higher rates of female adolescents (53.4%), 14-year-olds (45.8%), mixed ethnicity (42.0%), studying at public schools (76.0%), families with a low maternal education level (26.5%), living in the north and northeast region of the country (33.4%) and a low household assets and services score (33.4%). The data from the PeNSE 2015 edition show higher rates of male adolescents (50.6%), 16- and 17-year-olds (34.6%), mixed ethnicity (41.7%), studying at public schools (76.7%), families with a low maternal education level (25.3%), living in the north and northeast region (21.1%) and a low household assets and services score (33.5%) (Table 1).

**Table 1:** Description of the study population according to sociodemographic characteristics. PeNSE (2009–2015).

Variables	PeNSE 2009		PeNSE 2015	
	n	% (95% CI)	n	% (95% CI)
Sex				
Female	31,823	53.4 (52.9–53.7)	5,317	49.4 (48.4–50.3)
Male	27,814	46.6 (46.2–47.0)	5,453	50.6 (49.7–51.6)
Age				
13 years	15,051	25.3 (24.9–25.7)	2,524	23.4 (22.6–24.2)
14 years	27,243	45.8 (45.4–46.2)	2,111	19.6 (18.8–20.3)
15 years	11,141	18.7 (18.4–19.0)	2,408	22.4 (21.6–23.1)
16 years and older	6,035	10.2 (9.9–10.4)	4,648	34.6 (33.7–35.5)
Race/Skin color				
White	21,655	37.0 (36.7–37.4)	4,231	39.3 (38.4–40.2)
Black	7,323	12.5 (12.3–12.8)	1,267	11.8 (11.1–12.4)
Yellow	2,398	4.10 (3.9–4.3)	455	4.2 (3.8–4.6)
Mixed-race	24,556	42.0 (41.6–42.4)	4,493	41.7 (40.8–42.7)
Indigenous	2,560	4.40 (4.2–4.5)	316	2.94 (2.6–3.2)
School type				
Public	45,353	76.0 (75.7–76.4)	8,256	76.7 (75.8–77.4)
Private	14,284	24.30 (23.6–24.3)	2,514	23.3 (22.5–24.1)
Maternal education				
Non-educated/ Incomplete primary education	14,952	25.5 (25.1–25.8)	2,328	21.6 (20.8–22.4)
Completed primary education/ Incomplete secondary education	7,925	13.5 (13.2–13.8).	1,335	12.4 (11.8–13.01)
Completed secondary education/ Incomplete higher education	15,541	26.5 (26.1–26.8)	2,717	25.3 (24.4–26.0)
Completed higher education	10,265	17.5 (17.1–17.8)	2,032	18.9 (18.1–19.6)
Did not know	10,042	17.0 (16.8–17.4)	2,339	21.8 (20.9–22.5)
Region				
North	13,240	22.2 (19.0–22.5)	2,139	19.9 (19.1–20.6)
Northeast	19,941	33.4 (33.0–33.8)	2,277	21.1 (20.3–21.9)
Centre-West	9,837	16.5 (16.2–16.8)	2,119	19.7 (18.9–20.4)
Southeast	10,543	17.7 (17.4–18.0)	2,083	19.3 (18.6–20.1)
South	6,076	10.2 (9.9–10.4)	2,152	20.0 (19.2–20.7)
Household Assets and Services Score				
High	20,128	32.4 (32.1–32.8)	3,571	33.1 (32.3–34.0)
Medium	20,894	33.7 (33.3–34.0)	3,593	33.4 (32.5–34.5)
Low	21,053	33.9 (33.5–34.3)	3,606	33.5 (32.6–34.4)

The data further indicate significant differences between all of the studied sociodemographic predictors and the nutritional status of the adolescents in both the 2009 and 2015 editions of the PeNSE (p<0.05), except for sex and race/skin color that were not statistically significant in the PeNSE 2015 (Table 2).

**Table 2:** Analysis of associations between sociodemographic predictors and nutritional outcomes of adolescents. PeNSE (2009–2015).

Variables	PeNSE 2009					PeNSE 2015				
	Eutrophic	Overweight	Stunting	Double Burden of Malnutrition	p-value*	Eutrophic	Overweight	Stunting	Double Burden of Malnutrition	p-value*
	% (n)	% (n)	% (n)	% (n)		% (n)	% (n)	% (n)	% (n)	
**Sex**										
Female	76.9 (24,152)	20.4 (6,389)	2.2 (705)	0.5(140)	<0.001	73.6 (3,916)	24.2 (1,284)	1.8 (97)	0.4 (20)	0.081
Male	74.0 (20,288)	22.5 (6,164)	3.2 (877)	0.3 (74)		72.5 (3,953)	24.7 (1,347)	2.5 (136)	0.3 (17)	
**Age**					<0.001					<0.001
≤14 years	74.5 (31,163)	23.2 (9,683)	2.0 (854)	0.2 (99)		70.6 (3,273)	27.1 (1,256)	2.0 (94)	0.3 (12)	
>14 years	78.1 (13,277)	16.9 (2,870)	4.3 (728)	0.7 (115)		74.9 (4,596)	22.4 (1,375)	2.3 (1,369)	0.4 (25)	
**Race/ Skin color**					<0.001					0.167
White	73.8 (15,787)	27.7 (5,081)	2.2 (459)	0.3 (68)		72.0 (3,047)	25.5 (1,078)	2.1 (89)	0.4 (17)	
Non-white	76.7 (27,859)	20.0 (7,266)	2.9 (1,065)	0.4 (142)		73.8 (4,817)	23.7 (1,550)	2.2 (144)	0.3 (20)	
**Region**					<0.001					<0.001
South/Southeast/Centre-West	74.9 (19,520)	23.2 (6,039)	1.7 (453)	0.2 (56)		71.7 (4,552)	26.3 (1,673)	1.7 (106)	0.3 (23)	
North/Northeast	76.2 (2,920)	19.9 (6,514)	3.4 (1,129)	0.5 (158)		75.1 (3,317)	21.7 (2,631)	2.9 (127)	0.3 (14)	
**School type**					<0.001					<0.001
Private	70.0 (9,883)	28.7 (4,054)	1.1 (149)	0.2 (29)		70.2 (1,765)	28.7 (722)	0.8 (20)	0.3 (7)	
Public	77.4 (34,557)	19.0 (8,499)	3.2 (1,433)	0.4 (185)		73.9 (6,104)	23.1 (1,909)	2.6 (213)	0.4 (30)	
**Maternal Education**					<0.001					<0.001
Completed primary education/ Completed higher education	74.0 (24,638)	23.7 (7,881)	2.0 (670)	0.3 (92)		72.6 (4,415)	25.8 (1,572)	1.4 (82)	0.2 (15)	
Non-educated/ Incomplete primary education	78.1 (11,535)	17.4 (2,560)	3.9 (582)	0.6 (85)		73.9 (1,720)	22.4 (521)	3.3 (77)	0.4 (10)	
Did not know	76.9 (7,635)	19.8 (1,962)	3.0 (296)	0.3 (29)		73.5 (1,719)	22.9 (536)	3.1 (72)	0.5 (12)	
**Household Assets and Services Score**					<0.001					<0.001
High—3^0^ tercile	71.5 (14,208)	26.5 (5,278)	1.7 (332)	0.3 (59)		71.7 (2,560)	26.5 (946)	1.5 (55)	0.3 (10)	
Medium—2^0^ tercile	76.5 (15,778)	20.7 (4,280)	2.5 (512)	0.3 (71)		72.6 (2,608)	25.5 (918)	1.6 (57)	0.3 (10)	
Low—1^0^ tercile	79.1 (14,454)	16.4 (2,995)	4.0 (738)	0.5 (84)		74.9 (2,710)	21.3 (767)	3.3 (121)	0.5 (17)	

* Pearson’s χ^2^ test

The results of the multinomial logistic regression analysis indicate that in both periods of the PeNSE (2009–2015), the chance of overweight was lower, respectively, among adolescents over 14 years old [(OR = 0.78, 95% CI = 0.74–0.82), (OR = 0.90, 95% CI = 0.86–0.94)], residing in the north and northeast region [(OR = 0.89, 95% CI = 0.86–0.93), (OR = 0.79, 95% CI = 0.72–0.86)], from public schools [(OR = 0.76, 95% CI = 0.72–0.80), (OR = 0.82, 95% CI = 0.73–02.92)] and with the lowest household assets score [(OR = 0.72, 95% CI = 0.68–0.76), (OR = 0.87, 95% CI = 0.78–0.99)]. In both survey periods, the chance of stunting was higher, respectively, among boys [(OR = 1.49, 95% CI = 1.34–1.65), (OR = 1.38, 95% CI = 1.05–1.81)], those residing in the north and northeast region [(OR = 1.84, 95% CI = 1.64–2.07), (OR = 1.54, 95% CI = 1.17–2.02)] and those from public schools [(OR = 1.99, 95% CI = 1.64–2.41), (OR = 2.08, 95% CI = 1.28–3.39)] (Table 3).

**Table 3:** Multinomial logistic regression analysis between sociodemographic predictors and nutritional outcomes of adolescents. PeNSE (2009–2015).

Variables	PENSE 2009						PENSE 2015					
	Overweight		Stunting		Double Burden of Malnutrition		Overweight		Stunting		Double Burden of Malnutrition
	aOR	95% CI	aOR	95% CI	aOR	95% CI	aOR	95% CI	aOR	95% CI	aOR	95% CI
**Sex**												
Female	-	-	-	-	-	-	-	-	-	-	-	-
Male	**1.14**	**1.09–1.18**	**1.49**	**1.34–1.65**	**0.60**	**0.45–0.81**	1.03	0.94–1.13	**1.38**	**1.05–1.81**	0.83	0.43–1.61
**Age**												
≤14 years	-	-	-	-	-	-	-	-	-	-	-	-
>14 years	**0.78**	**0.74–0.82**	**1.55**	**1.39–1.72**	**2.40**	**1.80–3.20**	**0.90**	**0.86–0.94**	1.02	0.90–1.15	1.37	0.99–1.88
**Race/ Skin color**												
White	-	-	-	-	-	-	-	-	-	-	-	-
Non-white	**0.95**	**0.91–0.99**	1.00	0.90–1.13	0.94	0.69–1.28	1.04	0.91–1.20	0.89	0.59–1.32	0.40	0.96–1.68
**Region**												
South/Southeast/Centre-West	-	-	-	-	-	-	-	-	-	-	-	-
North/Northeast	**0.89**	**0.86–0.93**	**1.84**	**1.64–2.07**	**2.01**	**1.49–2.84**	**0.79**	**0.72–0.86**	**1.54**	**1.17–2.02**	0.85	0.43–1.67
**School type**												
Private	-	-	-	-	-	-	-	-	-	-	-	-
Public	**0.76**	**0.72–0.80**	**1.99**	**1.64–2.41**	1.49	0.94–2.36	**0.82**	**0.73–0.92**	**2.08**	**1.28–3.39**	0.89	0.36–2.20
**Maternal Education**												
Completed primary education/ Completed higher education	-	-	-	-	-	-	-	-	-	-	-	-
Non-educated/ Incomplete primary education	**0.89**	**0.84–0.94**	**1.38**	**1.22–1.56**	**1.48**	**1.07–2.04**	0.94	0.83–1.06	**1.91**	**1.37–2.70**	1.69	0.72–3.99
Did not know	0.92	0.87–0.98	1.23	1.06–1.42	0.91	0.59–1.40	0.90	0.79–1.01	1.91	1.36–2.69	2.18	0.95–5.0
**Household Assets and Services Score**												
High—3° tercile	-	-	-	-	-	-	-	-	-	-	-	-
Medium—2°tercile	**0.84**	**0.80–0.88**	1.11	0.96–1.29	0.83	0.57–1.20	0.99	0.88–1.10	0.90	0.62–1.33	0.95	0.39–2.30
Low—1° tercile	**0.72**	**0.68–0.76**	**1.35**	**1.16–1.57**	0.79	0.54–1.17	**0.87**	**0.78–0.99**	1.40	0.99–1.99	1.38	0.59–3.24

Reference category: normal BMI and height-for-age (BMI: Z-score ≤ +1 and H/A: Z-score -2);

aOR: adjusted odds ratio; 95%CI: 95% confidence interval;

aOR (95%CI) adjusted by all variables.

In the PeNSE 2009, the chance of DBM was lower among boys (OR = 0.60, 95% CI = 0.45–0.81) and higher among those over 14 years old (OR = 2.40, 95% CI = 1.80–3.20), those living in the north and northeast regions (OR = 2.01, 95% CI = 1.49–2.84) and from families with a low maternal education level (OR = 1.48, 95% CI = 1.07–2.04). In the 2015 edition, no significant associations were found for this outcome (Table 3).

## Discussion

This is a novel study aimed at measuring DBM and its socioeconomic determinants in the adolescent population of Brazil in 2009 and 2015. Despite the comparative limitations of the sample, it seems that overweight increased, stunting reduced and DBM seemed to be stable in the studied period. However, the DBM rates were well below those found in the study of Caleyachett et al. (2018) [14] who used health data from 57 LMICs between 2003 and 2013, which included 129,276 adolescents from 12 to 15 years old. In that study [14], the DBM phenomenon was found in 2.0% (95%CI = 1.7%, 2.5%) of the adolescents. The reason for this phenomenon is not yet fully understood: if there is sufficient energy for children to gain excess weight, then why do the children not reach their linear growth potential and remain stunted? Some studies suggest that this phenomenon is particularly related to inadequate nutrition during the period between conception, resulting in restricted intrauterine growth, and age below two years—which might entail retarded linear growth—followed by exposure to “obesogenic environments” at more advanced ages [21], [22], [8], [23], [3]. Other possible explanations for this phenomenon have been explored [23]. Some studies also suggest that retarded growth can change normal metabolic processes and thus increase insulin sensitivity and reduce fat oxidation, which increases the risk of excess weight gain at a later life stage [24], [25].

The results of the multinomial logistic regression analysis clearly show an increased prevalence of stunting among adolescents with an unfavourable socioeconomic status (in public school, from a family with a low maternal education level, from the north and northeast regions, and with worse household assets scores) in both evaluated editions (PeNSE 2009–2015). By contrast, the highest prevalence of overweight was found among adolescents from private schools, from families with a higher maternal education level, from the South, Southeast and Centre-West regions and in a more favourable economic situation. The effect of improved economic situation on the favourable trajectory of overweight/obesity is associated with the socioeconomic expansion and the processes of urbanization and globalization of the country, which result in negative changes in dietary and physical activity patterns [26]. These phenomena have been exhaustively discussed in the national [27] and international [28] literature in an isolated manner.

The main finding of the present study concerns the socioeconomic factors associated with the DBM phenotype [short and fat, according to Varela *et al*. (2012) [29]]. The data indicated higher chances of DBM among adolescents living under more adverse conditions. That is, DBM is positively associated with predictors of poverty and social inequality [23]. These more structuring factors are possibly acting through different chains of determination of both undernutrition and overnutrition, thus affecting the balance between the amount of nutrients and energy required by the body and between the amount of nutrients and energy consumed [30]. Many studies consider this phenomenon typical in economically developing countries that are passing through nutritional transition, such as Brazil and other LMICs [23]. The predominant feature of the nutritional transition in these regions is the epidemic emergence of overweight and, particularly, of obesity, as an event of greater epidemiological visibility and implications associated with the patterns of morbidity and mortality [31]. This has been accompanied by a decrease in deficiency diseases, such as stunting and micronutrient deficiencies, although these still persist in the scenario of morbidities in Brazil [6], [1].

Hence, health-related problems that are apparently of antagonistic origin occur simultaneously in the population. Thus, controlling DBM poses an important challenge for most developing countries [8]. Recently, the WHO published suggested actions to control DBM (which include interventions, programmes and policies) with the potential to simultaneously reduce the risk of undernutrition and overweight, obesity and diet-related NCDs [32]. One of the key principles of the WHO is to ensure that the actions planned to address one form of malnutrition do not unintentionally increase the risk of other form. These actions aim at providing easy access to healthy and nutritious foods and thereby to promote a healthy weight. This is also the objective of the United Nations Sustainable Development Goal (SDG) 2, which aims at ending hunger, ensuring safe, nutritious food and promoting sustainable agriculture [33].

### Limitations

Since the PeNSE 2009 and 2015 are cross-sectional surveys, this study has some limitations such as the determination of causality. Some precaution is necessary in the comparison of results between the editions, considering the changes sampling plans of the PeNSE. In 2009, the target-population of PeNSE was formed by students enrolled in the 9th grade of Elementary School; in 2015, students enrolled from the 6th grade of elementary school to the 3rd grade of high school were also included with the objective of having a more representative research for students aged from 13 to 17 years old. Furthermore, it is important to note that the sampled population excluded young individuals who are not in school. Nevertheless, the school-based methodology has been adopted in European countries and worldwide because of the easy access to the adolescent population and because of the benefits that arise from the study, which enable integrated planning by the health and education sectors for the target population. Furthermore, the coverage of the educational system in Brazil has increased, thus approaching universalisation. Additionally, the fact that the survey sampled two strata (public and private school students) extends the representativeness of the target population. A further limitation is the fact that the questionnaire used to obtain the household assets and services score is self-reported, being then subject to information bias. The use of BMI as a measure of adiposity among adolescents also seems to be a further limitation of the study. According to many researchers, BMI must be used specially in epidemiological studies since it requires only the collection of simple anthropometric measures that are non-invasive, easily collected, low-cost and well-accepted by the general population [34]. However, its interpretation as an index of adiposity must be made with caution. Nevertheless, we believe that our results are consistent for the following reasons. First, the PeNSE is the widest-ranging survey of students in Brazil, in terms of both the sample size and the surveyed topics. The data from the different editions of the PeNSE are considered high-quality because they were obtained by following standardised procedures, and, in most cases, such surveys are the only source of data on student health that some countries have for surveillance, particularly in developing economies and those in transition. Second, in general, the findings corroborate those of similar studies and were analysed using appropriate statistical methods.

## Conclusion

The results of the PeNSE 2009 and 2015 indicate the epidemic emergence of overweight and a reduction in stunting, although it still persists as a morbidity among the poorest adolescents of the country. Cases where both conditions (overweight and stunting) are simultaneously present in the same individual characterise DBM, which was observed at low rates in both of the studied survey editions. The results from the PeNSE 2009 also indicate the presence of socioeconomic inequalities in the occurrence of DBM and the remaining studied outcomes. Undoubtedly, the social determinants of health have a strong influence on the opportunities and the ways Brazilians eat and on the risks related to the nutritional status of individuals and communities, which indicates a need for answers beyond the health sector. Thus, we suggest that simultaneous interventions in the area of equity be conducted to prevent the advancement of nutritional problems.
