# Correction: Factors associated with the double burden of malnutrition among adolescents, National Adolescent School-Based Health Survey (PENSE 2009 and 2015)

**DOI:** 10.1371/journal.pone.0219315

**Published:** 2019-06-27

**Authors:** Júlia Caffé Oliveira Uzêda, Rita de Cássia Ribeiro-Silva, Natanael de Jesus Silva, Rosemeire L. Fiaccone, Débora C. Malta, Naiá Ortelan, Maurício L. Barreto

The seventh author's name is spelled incorrectly. The correct name is: Maurício L. Barreto
